# Environmental effects on stem water deficit in co-occurring conifers exposed to soil dryness

**DOI:** 10.1007/s00484-014-0853-1

**Published:** 2014-05-29

**Authors:** Walter Oberhuber, Werner Kofler, Roman Schuster, Gerhard Wieser

**Affiliations:** 1 Institute of Botany, Leopold-Franzens-University of Innsbruck, Sternwartestrasse 15, 6020 Innsbruck, Austria; 2 Department of Alpine Timberline Ecophysiology, Federal Research and Training Centre for Forests, Natural Hazards and Landscape (BFW), Rennweg 1, 6020 Innsbruck, Austria

**Keywords:** Conifers, Dendrometer, Drought, Stem radius variation, Stem water deficit

## Abstract

We monitored dynamics of stem water deficit (Δ*W*) and needle water potential (*Ψ*) during two consecutive growing seasons (2011 and 2012) in a dry inner Alpine environment (750 m above sea level, Tyrol, Austria), where *Pinus sylvestris*, *Picea abies* and *Larix decidua* form mixed stands. Δ*W* was extracted from stem circumference variations, which were continuously recorded by electronic band dendrometers (six trees per species) and correlations with environmental variables were performed. Results revealed that (i) Δ*W* reached highest and lowest values in *P. abies* and *L. decidua*, respectively, while mean minimum water potential (*Ψ*_ea_) amounted to −3.0 MPa in *L. decidua* and −1.8 MPa in *P. abies* and *P. sylvestris*. (ii) Δ*W* and *Ψ*_ea_ were significantly correlated in *P. abies* (*r* = 0.630; *P* = 0.038) and *L. decidua* (*r* = 0.646; *P* = 0.032). (iii) In all species, Δ*W* reached highest values in late summer and was most closely related to temperature (*P* < 0.001). Results indicate that all species were undergoing water limitations as measured by increasing Δ*W* throughout the growing season, whereby *P. abies* most strongly drew upon water reserves in the living tissues of the bark. Quite similar Δ*W* developed in drought-sensitive *L. decidua* and drought-tolerant *P. sylvestris* indicate that various water storage locations are depleted in species showing different strategies of water status regulation, i.e. anisohydric vs. isohydric behavior, respectively, and/or water uptake efficiency differs among these species. Close coupling of Δ*W* to temperature suggests that climate warming affects plant water status through its effect on atmospheric demand for moisture.

## Introduction

Although plant water status is determined mainly by the physical conditions of the air and soil, tissue water storage in trees was found to be an important factor in transiently regulating tree water relations (e.g. Meinzer et al. 2004; Čermak et al. 2007). Several water storage locations within the tree exist, namely, the sapwood, the cell walls and inactive vessels and the living cells, i.e. elastic tissues of the bark (i.e. cambium, phloem and parenchyma) and mesophyll of needles (Zimmermann 1983). Water in these storage locations is partly depleted and replenished daily by changing water potential gradients within the plant (Whitehead and Jarvis 1981). Changes in daily water content of the stem together with irreversible radial growth, i.e. cambial cell division and enlargement, and temperature expansion explain stem radius variations during the growing season, whereby Zweifel et al. (2001) reported that the influence of temperature on daily stem shrinkage and swelling is negligible. Stem radius variations detrended for growth were called tree water deficit (Δ*W*) by Hinckley and Lassoie (1981), and this parameter was found to be proportional to water content in the living tissues of the bark (Herzog et al. 1995). According to several authors (Zweifel et al. 2005; Drew et al. 2011; Köcher et al. 2013), Δ*W* can be interpreted as a direct measure of drought stress in trees and has been shown to be primarily determined by atmospheric environment and soil water content (SWC, e.g. Zweifel et al. 2005; Ehrenberger et al. 2012). Dendrometer measurements of stem radius variations reveal small changes in stem water content with high temporal resolution and automated dendrometers are a less labor-intensive method to record intra-annual dynamics of plant water relations compared to measuring leaf water potential (*Ψ*).

Scots pine (*Pinus sylvestris* L.) is usually the dominating tree species at xeric sites in dry inner Alpine valleys and is well known as a drought-tolerant species (e.g. Weber et al. 2007). At dry-mesic sites, it co-occurs with competitor species (Norway spruce, *Picea abies* (L.) Karst. and European larch, *Larix decidua* Mill.). *P. abies* is most widespread in Central European Alps ranging from low elevation up to timberline, while *L. decidua* is more common at higher elevation (Ellenberg and Leuschner 2010). These co-occurring conifers show different successional and phenological traits, whereby evergreen *P. sylvestris* and deciduous *L. decidua* are light-demanding species dominating in early successional stages, while evergreen *P. abies* is a moderately shade-tolerant tree, which predominates in the late successional stage (Ellenberg and Leuschner 2010). Swidrak et al. (2013) found that growth resumption after winter dormancy differed by several weeks between early successional *L. decidua* and *P. sylvestris* and late successional *P. abies*, whereby earlier onset of aboveground growth in *L. decidua* and *P. sylvestris* supports the hypothesis that pioneer species adopt riskier life strategies compared to late successional *P. abies* (Körner 2006). Shade tolerance and shallow rooting were suggested to allow *P. abies* to invade *P. sylvestris* stands at dry-mesic sites (Schuster and Oberhuber 2013b) and Anfodillo et al. (1998) found that *L. decidua* is very efficient at adapting to drought by osmotic adjustment.

Previous dendroecological and dendroclimatological studies within the dry inner Alps revealed that radial growth of these conifers is primarily limited by low water availability in spring and high air temperature in early summer (e.g. Oberhuber et al. 1998; Rigling et al. 2002; Schuster and Oberhuber 2013a). High vulnerability of *P. abies* and *L. decidua* to soil water deficits especially of trees growing on mesic sites was also reported by Lévesque et al. (2013). Eilmann and Rigling (2013) also regard *L. decidua* to be maladjusted to dry conditions, which might be related to its anisohydric strategy, i.e. high transpiration rates are maintained even under conditions of low water availability, while *P. sylvestris* and *P. abies* stabilize their water relations by closing stomata early (isohydric behaviour; Anfodillo et al. 1998; Leo et al. 2013). Köcher et al. (2013) suggested that the functional significance of water storage differs among tree species when their wood density, hydraulic architecture and strategies of water status regulation, i.e. isohydric vs. anisohydric behaviour, are different.

The aims of this study therefore were (i) to compare seasonal development of Δ*W* and *Ψ* in co-occurring conifers (deciduous *L. decidua*, evergreen *P. abies* and *P. sylvestris*) exposed to soil dryness during the growing season and (ii) to determine environmental factors which contribute most to the overall variations in Δ*W*. We hypothesized that (i) during the growing season, most distinct Δ*W* is developed in *P. abies* due to its low adaptability to drought-prone conditions and (ii) Δ*W* is most closely related to climate variables which influence transpiration.

## Materials and methods

### Study area

**Fig. 1:**
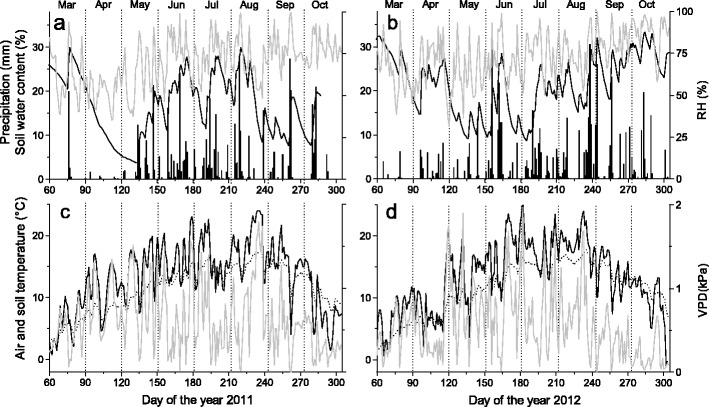
Climate variables, soil water content (Vol. %) and soil temperature recorded during the growing seasons 2011 and 2012 within the study plot. **a**, **b** Daily precipitation sum (*bars*), soil water content in 5–10 cm soil depth (*black line*) and relative air humidity (*RH*, *grey line*). **c**, **d** Mean daily air (*black line*) and soil temperature (*dotted line*) recorded above canopy and in 5–10 cm soil depth, respectively, and calculated vapour pressure deficit (*VPD*, *grey line*)

The study site is part of a postglacial rock-slide area situated in the montane belt [ca. 750 m above sea level (a.s.l.)] within the inner Alpine dry valley of the Inn River (Tyrol, Austria, 47°13′53″N, 10°50′51″E). Annual mean air temperature and total precipitation amount to 7.3 °C and 716 mm, respectively (long-term mean during 1911–2010 at Ötz, 812 m a.s.l., 5 km from the study area). Although the dominating plant community is an open Spring Heath-Pine wood (*Erico-Pinetum typicum*), on scattered dry-mesic sites mixed stands composed of *P. sylvestris* (60 %), *P. abies* (20 %) and *L. decidua* (20 %) are developed. At these sites a thick moss layer occurs in the understorey, indicating slightly moist conditions. Soil water content in 5–10 cm soil depth ranged from 0.05 to 0.30 m^3^ m^−3^ during April through September (cf. Fig. 1). Shallow soils of protorendzina type, i.e. rendzic leptosols according to the FAO classification system (FAO 2006), are developed and consist of unconsolidated, coarse-textured materials with low water-holding capacity (soil depth 10–20 cm). Tree height and canopy coverage of the selected stand were 15 to 18 m and ca. 70 %, respectively. The study site was slightly facing north (slope angle 5°) in some parts. Mean tree age and diameter at breast height ranged from 115 years (*P. abies*) to 150 years (*P. sylvestris*, *L. decidua*) and 23.0 cm (*P. abies*) to 27.8 cm (*P. sylvestris*, *L. decidua*), respectively (cf. Schuster and Oberhuber 2013a).

### Dendrometer records

In autumn 2010, we installed temperature compensated electronic band dendrometers (DC2, Ecomatik, Munich, Germany) with a resolution of 2 μm at six dominant trees per species to measure diurnal circumference variation at breast height (1.3 m). The temperature coefficient of the sensor amounted to <0.1 μm/K. The measuring cable consisted of Invar steel, which shows a temperature coefficient of linear expansion < 1 μm/mK. Individual trees were randomly selected, but trees with major stem or crown anomalies were excluded. Dead outermost layers of the bark (periderm) were slightly removed to reduce the influence of hygroscopic swelling and shrinkage of the bark on dendrometer records (DMR) and to ensure close contact with the stem. Data loggers were programmed to record measurements taken every 30 min, and daily increment of stem radius was calculated by averaging all daily measurements (48 values/day). Data were recorded in Central European Summer Time, i.e. Coordinated Universal Time (UTC) +2:00. Determination of the baseline in DMR can be masked by water-related swelling or shrinkage of the stem caused by stem rehydration after frost-induced shrinkage during winter and stem dehydration during drought periods, respectively (Turcotte et al. 2011). In previous studies, histological analyses of developing tracheids allowed proper timing of radial growth onset in dendrometer traces of conifers exposed to drought (Oberhuber and Gruber 2010). Similarly, dendrometer traces in this study were set to zero at the day of the year, when first row of enlarging cells was detected.

### Tree water deficit

**Fig. 2:**
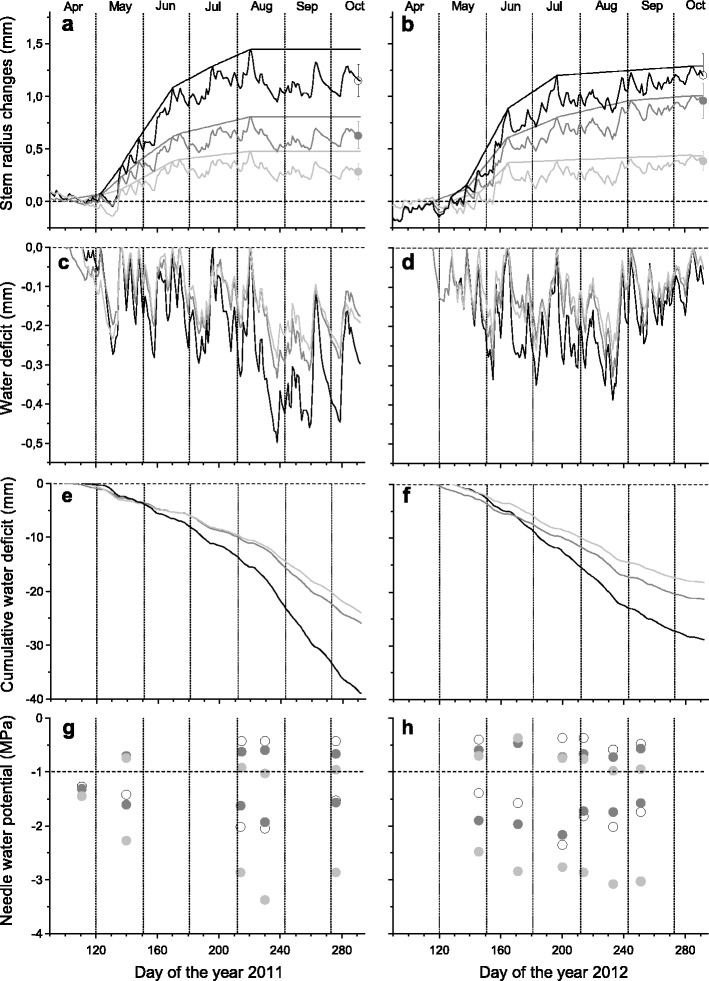
Time series of mean daily dendrometer records and growth-trend lines over-bark (**a**, **b**), mean daily extracted (**c**, **d**) and cumulative stem water deficit (Δ*W*) (**e**, **f**) and maximum (*Ψ*_pd_) and minimum (*Ψ*_ea_) needle water potential (**g**, **h**; *Ψ*_pd_ and *Ψ*_ea_ are separated by *dashed line* at −1 MPa). Species are denoted by *solid line and open circle* (*Picea abies*), *dark grey line and filled circle* (*Pinus sylvestris*) and *light grey line and filled circle* (*Larix decidua*). Bars in **a** and **b** represent standard deviations among dendrometer records (*n* = 6)

To separate water storage-related stem radius changes in the living tissues of the bark from radial growth, dendrometer records were de-trended for growth according to Zweifel et al. (2005). In particular, from the maximum value in the dendrometer data (*D*_max_) a horizontal line was drawn to the end of the data set, i.e. to the right. In the following, the slope of the line was increased past *D*_max_, i.e. to the left, until it touched the next maximum value. This procedure was repeated until the earliest data point was reached. By applying this method, it is assumed that radial growth occurred constantly throughout the growing season, i.e. including periods of stem radius contraction, rather than being restricted to short periods where the stem is rehydrated (cf. Fig. 2a–b). Tree water deficit (Δ*W*) was calculated as the difference in stem size under increasingly dry conditions relative to the stem size under fully hydrated conditions (Hinckley and Lassoie 1981). By removing the over-bark growth trend, a measure of water influx- and efflux-related changes in stem size is obtained, which may be interpreted with caution as stem water status. Hence, a zero value of Δ*W* indicates fully hydrated conditions, while increasingly negative numbers indicate increasing drought stress. Because diurnal changes in stem radius are related mainly to changes in the elastic living tissues of the bark (e.g. Daudet et al. 2005), increment cores were taken during a cool moist period in fall to determine bark width (excluding periderm).

### Climate influence on tree water deficit

Time series of environmental variables [precipitation, relative air humidity (RH), vapour pressure deficit (VPD), air and soil temperature, soil water content (SWC)] were compared with daily time series of Δ*W* using Pearson product–moment correlation statistics for the period April–September. Kolmogorov–Smirnov tests were applied to check for normal distribution of selected variables. Kendall’s rank correlation coefficient (*τ*, Sheskin 2007) was determined for not normally distributed variables. The software package used for the analysis was PASW Statistics 18, version 18.0.2. (IBM, NY, USA).

### Needle water potential

Leaf water potential (*Ψ*) was monitored during the growing season 2011 and 2012 at pre-dawn (*Ψ*_pd_), early afternoon (*Ψ*_ea_) and late afternoon (*Ψ*_la_) on warm sunny days using a pressure chamber (Model 1000; PMS Instrument, Corvallis, OR, USA). *Ψ* was recorded on May 20, August 2, 3, 18 and 19 and October 3 and 19 in 2011 and on May 25 and 26, June 19, July 18, August 1 and 20 and September 7 in 2012. Because *Ψ*_la_ was not determined in September and October, and some measurements had to be discarded due to clouding, the number of observations used in analyses was 11 for pre-dawn and early afternoon and 6 for late afternoon. A scaffold of 16 m height was constructed to determine daily variation in *Ψ* in the upper crown of the same trees used to trace growth phenology (Swidrak et al. 2013). One-year-old twigs of *P. sylvestris* and *P. abies* and current year twigs of *L. decidua* were cut from the canopy and *Ψ* was measured within 5 min of sampling. *Ψ* of three samples per species and time was determined and mean values were calculated.

### Microclimate records

During the study period, air temperature, relative air humidity (RH) and daily precipitation were collected automatically (ONSET, Pocasset, MA, USA) above canopy at top of a scaffold at ca. 18 m height. Measuring intervals for all sensors were 30 min and mean daily air temperatures were calculated by averaging all measurements (48 values/day). Vapour pressure deficit in the air (VPD) was calculated from the hourly means of air temperature and RH using the equation given in Prenger and Ling (2000). Volumetric soil water content (SWC) in 5–10 cm soil depth was recorded within the study plot (ThetaProbes Type ML2x, Delta-T, Cambridge, England). Additionally, soil temperature in the top 5–10 cm soil depth was measured (HOBO, ONSET, Pocasset, MA, USA). Measuring intervals were set to 60 min and mean daily SWC (vol. %) and soil temperature (Celsius) were calculated by averaging all measurements from three sensors.

### Environmental variables during growing seasons 2011 and 2012

Climate in 2011 and 2012 distinctly deviated at the start of the growing season in spring. An almost continuous drought period lasted from 19 March to 13 May 2011 (Fig. 1a), which caused SWC at the study plot to drop to ca. 5 vol. % in early May. Starting with rainfall events in mid-May 2011 SWC reached 20 to 30 vol. % until mid-August, when low rainfall caused a decrease of SWC to ca. 10 vol. % for several weeks (Fig. 1a). Rainfall was more evenly distributed over the growing season in 2012 (Fig. 1b). In 2012, frequent rainfall events in March and April caused high SWC (ca. 25 vol. %) until May, when SWC temporarily dropped to 10 vol. % due to low rainfall and abruptly increasing temperature (Fig. 1b). Daily mean air temperatures in April and May were 3.2 and 0.6 °C higher in 2011 (Fig. 1c) compared to 2012 (Fig. 1d). Air temperature and rainfall during summer 2012 exceeded records in 2011 by 1.2 °C and 86 mm, respectively. Daily mean soil temperature in 5–10 cm soil depth generally followed trend in air temperature but showed minor amplitudes (Fig. 1c, d). Mean VPD during May–August amounted to 0.7 kPa in both study years. VPD maxima reached 1.8 and 2 kPa in 2011 and 2012, respectively.

## Results

### Stem radius changes and dendrometer-derived tree water deficit

Calculated Δ*W* of all species showed synchronous fluctuations in both study years (Fig. 2a–d). During dry periods prior to about day 215, Δ*W* ranged between −0.2 and −0.3 mm. In mid-August 2011, Δ*W* abruptly dropped to ca. −0.5, −0.35 and −0.3 mm in *P. abies*, *P. sylvestris* and *L. decidua*, respectively, which did not recover by mid-October in all species indicating a permanent stem water deficit. Throughout both growing seasons, highest values of Δ*W* were developed in *P. abies* reaching ca. −0.5 mm in August 2011 (Fig. 2c, d). In 2012, Δ*W* reached highest values in mid-August ranging from −0.25 to −0.4 mm, which recovered with heavy rainfall events occurring in autumn. Cumulative Δ*W* reached highest values in *P. abies* and lowest values in *L. decidua* in both study years (Fig. 2e, f). Widths of living tissues of the bark amounted to 1.61 ± 0.35 mm in *P. sylvestris*, 3.59 ± 0.53 mm in *P. abies* and 3.22 ± 0.55 mm in *L. decidua*.

**Fig. 3:**
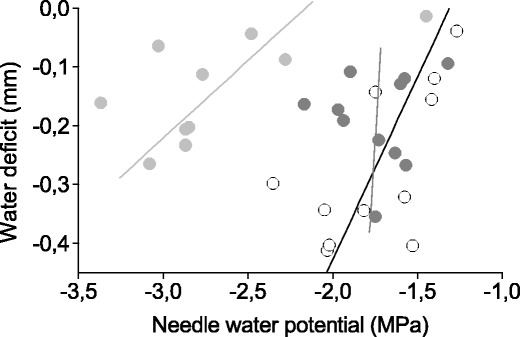
Relationship between stem water deficit (Δ*W*) and minimum needle water potential (*Ψ*_ea_). Δ*W* was determined at the same time of the day, when *Ψ*_ea_ were measured during the growing seasons 2011 and 2012. Species are denoted by *solid line and open circle* (*Piceaabies*), *dark grey line and filled circle* (*Pinus sylvestris*) and *light grey line and filled circle* (*Larix decidua*)

*Ψ*_pd_ did not drop below −0.75 in *P. abies* and *P. sylvestris* (Fig. 2g, h). During both growing seasons, *Ψ*_pd_ was predominantly lowest in *L. decidua* and slightly dropped below −1.0 MPa in August 2011 (−1.03 MPa). Mean *Ψ*_pd_ during both growing seasons ranged from −0.48 MPa in *P. abies* to −0.85 MPa in *L. decidua* (Table 1). Afternoon minimum water potentials (*Ψ*_ea_) dropped to −2.35 MPa in *P. abies*, −2.17 MPa in *P. sylvestris* and −3.37 MPa in *L. decidua*. At all sampling dates, latter species reached lowest *Ψ*_ea_ during both growing seasons, and mean *Ψ* were significantly lower throughout the day compared to other species (*P* < 0.01; Fig. 2g–h; Table 1). In all species, *Ψ* slightly recovered in late-afternoon (Table 1). Linear relationships between Δ*W* and *Ψ*_ea_ are depicted in Fig. 3. Statistically significant (*P* < 0.05) Pearson correlation coefficients were found for *L. decidua* (*y* = 3.796*x* − 2.162; *r* = 0.646; *P* = 0.032) and *P. abies* (*y* = 1.611*x* − 1.311; *r* = 0.630; *P* = 0.038), whereby in the former species, Δ*W* decreased more than twice as fast with increasing *Ψ*_ea_ as in the latter species (Fig. 3). Δ*W* and *Ψ*_ea_ were not significantly correlated in *P. sylvestris* (*y* = 0.214*x* − 1.701; *r* = 0.072; *P* = 0.834).

**Table 1:** Mean leaf water potential (*Ψ*) measured during growing seasons 2011 and 2012 throughout the day

	Mean leaf water potential (Megapascal)		
	*Ψ*_pd_ (6 a.m.)	*Ψ*_ea_ (2 p.m.)	*Ψ*_la_ (6 p.m.)
*Picea abies*	−0.48 ± 0.12^a^	−1.85 ± 0.30^a^	−1.69 ± 0.25^a^
*Pinus sylvestris*	−0.65 ± 0.08^b^	−1.77 ± 0.21^a^	−1.69 ± 0.14^a^
*Larix decidua*	−0.85 ± 0.22^c^	−2.98 ± 0.28^b^	−2.73 ± 0.26^b^

Data are means ± standard deviation. *n* = 11 for pre-dawn (*Ψ*_pd_) and early afternoon (*Ψ*_ea_) and 6 for late afternoon (*Ψ*_la_) measurements. Statistically significant differences of mean values between species are labelled by different letters (*P* ≤ 0.01; Student’s *t* test for independent samples)

### Influence of environmental variables on tree water deficit

**Fig. 4:**
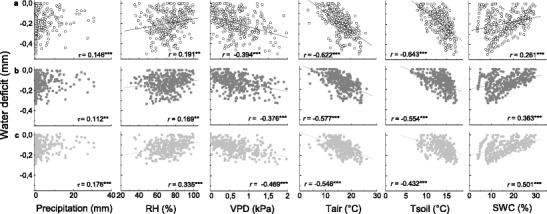
Correlations between stem water deficit (Δ*W*) of *Picea abies* (**a** *open circles*), *Pinus sylvestris* (**b** *dark grey filled circles*) and *Larix decidua* (**c** *light grey filled circles*) and environmental variables [precipitation, relative air humidity (*RH*), vapour pressure deficit (*VPD*), air temperature (*T*_*air*_), soil temperature (*T*_*soil*_) and soil water content (*SWC*)] for the period April–September 2011 and 2012. Pearson’s correlation coefficient (*r*) and Kendall’s tau coefficient (*τ*) were calculated (*n* = 365; ^**^ *P* < 0.01; ^***^ *P* < 0.001)

**Fig. 5:**
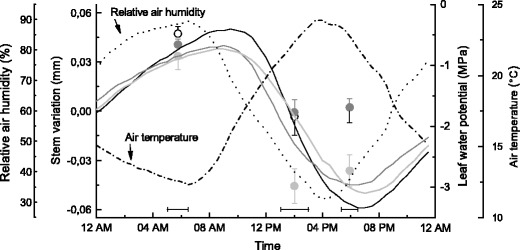
Diurnal cycle of standardized stem radius variation (half-hourly records minus daily mean) and leaf water potential (*Ψ*, mean values ± standard deviation) compared to relative air humidity and air temperature. Stem radius variations and climate variables were averaged from daily records when *Ψ* was determined. Species are denoted by *solid line and open circle* (*Picea abies*), *dark grey line and filled circle* (*Pinus sylvestris*) and *light grey line and filled circle* (*Larix decidua*). Horizontal bars indicate standard deviation with respect to measurements of *Ψ*

In Fig. 4a–c, Pearson product–moment correlations between Δ*W* and environmental variables are depicted for selected species. Strongest relationships were found with air and soil temperature and VPD (*P* < 0.001). Δ*W* was also significantly related to SWC and RH. Lowest coefficients (Kendall’s *τ*) were observed between Δ*W* and precipitation in all species. That diurnal stem variation followed air temperature and RH is depicted in Fig. 5. A lag effect of ca. 3 h between daily maximum/minimum and minimum/maximum values of RH and air temperature, respectively, and diurnal stem variation maximum/minimum values is obvious. In all species, the onset of contraction started at ca. 10:00 a.m. and that of expansion at ca. 5:00 p.m. The greatest amplitude in diurnal stem variation was detected in *P. abies* (109 μm), while approximately 20 % lower and similar amplitudes amounting to 85 and 88 μm were found in *P. sylvestris* and *L. decidua*, respectively (Fig. 5).

## Discussion

The water status of plants can be measured by direct methods (e.g. relative water content; leaf water potential) and indirect methods (e.g. stem, leaf or fruit shrinkage; reduction in cell expansion, growth and photosynthesis rate; Jones 2007). Hence, Δ*W* determined in this study by detrending dendrometer records for growth represents an indirect measure of plant water status. Advantages of the applied method are that (i) dendrometers allow a continuous, nondestructive record of plant water status, and (ii) Δ*W* was determined at the site of the process of interest, i.e. radial stem growth. Because all measures of tissue water status including leaf water potential are subject to homeostatic regulation, i.e. stomatal control tends to maintain water status stable especially in isohydric plants (Jones 2007), Δ*W* does not represent the ‘real’ water deficit in selected conifers. Indirect measures of water status, however, are regarded to be very valuable for detecting physiological responses to water deficits (Naor and Cohen 2003; Zweifel et al. 2005; Jones 2007).

### Seasonal development of stem water deficit

Despite the fact that trees were exposed to similar microclimate conditions above and below ground, differences in seasonal development of Δ*W* were found among co-occurring coniferous species. In accordance with our first hypothesis, *P. abies* showed highest seasonal Δ*W* in both years, and the amplitude of daily stem variation exceeded those of *P. sylvestris* and *L. decidua*. Latter findings are in accordance with data of King et al. (2013) investigating stem radius variation in *P. abies* and *L. decidua* with respect to similar timing for the beginning of stem water use (i.e. onset of stem contraction) and greater amplitudes in *P. abies* compared to *L. decidua*. The lag observed between environmental variables and onset of water depletion and replenishment in the living tissues of the bark in the morning and evening, respectively, indicates a time lag between water loss by transpiration in the canopy and use and refilling of water reserves at 1.3 m stem height (Meinzer et al. 2004). The significant relationship found between Δ*W* and minimum needle water potential (*Ψ*_ea_) indicates that transpiration strongly draws upon water reserves from the living bark. Zweifel et al. (2001) suggested that the utilization of internal stem water reserves makes *P. abies* in the short term less dependent on soil water content and prevents low stem water potentials that might be caused by peaks of transpiration. On the other hand, throughout the growing season, highest *Ψ*_pd_ was found in *P. abies*, indicating continuous rehydration of needles overnight. Most likely, the shallow root system of *P. abies* is most efficient in water uptake from upper soil layers, where small rain events increase soil water content. This finding might also explain why *P. abies* is able to temporarily invade *P. sylvestris* stands at dry-mesic sites within the study area, which was previously reported by Schuster and Oberhuber (2013b).

In all species, Δ*W* amounted to less than −0.1 mm at the time of growth resumption in spring (cf. Oberhuber et al. 2014) and reached maximum values in late summer when highest temperatures were recorded. High evapourative demand and long day length most likely reduced the duration of the recovery phase, i.e. an insufficient replenishment of water in the expandable tissues led to long-term increases in Δ*W*. Our findings are in agreement with reports of Vieira et al. (2013), who detected a markedly increase in Δ*W* in Mediterranean pine (*P. pinaster*) during summer. Seasonal increase of Δ*W* might be related to early decrease in aboveground growth reported within the study area (Oberhuber et al. 2014), because a shift in carbon allocation to roots in response to drought is a well-known phenomenon (e.g., Brunner et al. 2009). Our reasoning is supported by findings that latewood formation is adversely affected by occurrence of summer drought within the study area (Pichler and Oberhuber 2007) and elsewhere (e.g., Levanič et al. 2009).

In *P. sylvestris* and *P. abies*, a stomatal control of tree water status is indicated by the finding that Δ*W* values of −0.3 mm corresponded to *Ψ*_ea_ of ca. −1.75 MPa, while in *L. decidua*, a corresponding value of < −3 MPa was detected. By this way, *P. sylvestris* and *P. abies* maintain minimum *Ψ* at a significantly higher level than *L. decidua* and therefore avoid risk of hydraulic failure during periods of high evapourative demand. A water-saving strategy, i.e. isohydric behaviour of *P. sylvestris* and *P. abies* is in accordance with findings of Leo et al. (2013) within the study area that under artificial drought, stress sap flow density in *P. sylvestris* and *P. abies* was strongly reduced. Furthermore, although *P. sylvestris* is known as a drought-tolerant species (Weber et al. 2007; Ellenberg and Leuschner 2010), Zweifel et al. (2009) reported that *P. sylvestris* needs rain at regular intervals to keep its stomata open and has limited ability to withdraw stored water from its stem. This is consistent with most narrow bark width (excluding periderm) found in *P. sylvestris* amounting to ca. 50 % compared to co-occurring species. That species-specific differences in size of the shrinking tissue and differences in the conductance of water between the bark and the xylem vessels can affect dynamics of Δ*W* was reported by, e.g., Waring et al. (1979) and Naor and Cohen (2003). On the other hand, the finding that least Δ*W* was developed in *L. decidua* might be due to its anisohydric behaviour, i.e. high transpiration rates are maintained irrespective of soil water availability (Anfodillo et al. 1998; Leo et al. 2013). Hence, lowest *Ψ* found in *L. decidua* may allow sufficient water extraction from the soil, but results of Schulze et al. (1985) have to be considered, who reported that a *L. decidua* × *L. kaempferi* hybrid stored twice as much water in stem sapwood as *P. abies*. On the other hand, although *L. decidua* is characterized as a deep-rooted species (Kutschera and Lichtenegger 2002), access to ground water within the study area can be excluded from soil coring down to 100 m below ground, because no ground water table could be detected (Austrian Federal Railways, personal communication).

### Environmental control of tree water deficit

Results of this study revealed that Δ*W* of conifers exposed to soil dryness was more strongly controlled by air temperature than precipitation or SWC, which confirms our second hypothesis that Δ*W* is related to climate variables which influence transpiration. Furthermore, although air and soil temperature are inter-correlated, soil temperature is known to be important for water transported along the soil–plant–atmosphere continuum. In cold soils, increased water viscosity and decreased root permeability and fine-root growth are known to limit water uptake in conifers (Kramer and Boyer 1995). That SWC affected Δ*W* of *P. sylvestris* and *L. decidua* more strongly than Δ*W* of *P. abies* most likely indicates that in the short term, the use of water stored in the living tissues of the bark makes *P. abies* less dependent on the current water availability in the soil, which is consistent with results of Turcotte et al. (2011) and King et al. (2013). Coupling of Δ*W* to atmospheric conditions indicates that increasing temperature due to climate warming can affect plant water status through its effects on VPD, which increases exponentially when temperature increases (Breshears et al. 2013). However, stomatal conductance, which is the most important mechanism by which plants control water loss during periods of high evapourative demand, is partially regulated by internal factors (biochemical messengers, tissue water status), and the same individual can switch from an isohydric behaviour when SWC is low to an anisohydric behaviour when SWC is high (Domec and Johnson 2012 and references therein). Additionally, relocation of water from other storage locations (sapwood, cell walls and inactive vessels) to the elastic tissues of the bark might contribute to decoupling of environmental factors from Δ*W*.

## Conclusions

Our results indicate that all selected species were undergoing water limitations as measured by increasing Δ*W* throughout the growing season, which is in accordance with previous findings that soil water availability limits tree growth within the study area (e.g., Schuster and Oberhuber 2013a). Low water-holding capacity of the shallow stony soils, which causes fast decrease of SWC during rainless periods, makes our study area comparable to other dry inner Alpine environments, where a high vulnerability of *P. abies* and *L. decidua* to drought was determined by Lévesque et al. (2013). In line with their results, we found highest Δ*W* in *P. abies*, but quite similar Δ*W* was developed in drought-sensitive *L. decidua* and drought-tolerant *P. sylvestris* during both study years, which most likely indicates that different water storage locations are depleted when high evapourative demand prevails and/or water uptake efficiency at the drought-prone site differs among species.
